# Metabolic Dysfunction in Myalgic Encephalomyelitis/Chronic Fatigue Syndrome Not Due to Anti-mitochondrial Antibodies

**DOI:** 10.3389/fmed.2020.00108

**Published:** 2020-03-31

**Authors:** Isabell Nilsson, Jeremy Palmer, Eirini Apostolou, Carl-Gerhard Gottfries, Muhammad Rizwan, Charlotte Dahle, Anders Rosén

**Affiliations:** ^1^Division of Cell Biology, Department of Biomedical and Clinical Sciences, Linköping University, Linköping, Sweden; ^2^The Medical School, The University Newcastle upon Tyne, Newcastle upon Tyne, United Kingdom; ^3^The Gottfries Clinic AB, Mölndal, Sweden

**Keywords:** myalgic encephalomyelitis/chronic fatigue syndrome, anti-pyruvate dehydrogenase complex antibodies, PDC, anti-mitochondrial autoantibodies, AMA

## Abstract

Metabolic profiling studies have recently indicated dysfunctional mitochondria in myalgic encephalomyelitis/chronic fatigue syndrome (ME/CFS). This includes an impaired function of pyruvate dehydrogenase complex (PDC), possibly driven by serum factor(s), which leads to inadequate adenosine triphosphate generation and excessive lactate accumulation. A reminiscent energy blockade is likely to occur in primary biliary cholangitis (PBC), caused by anti-PDC autoantibodies, as recently proposed. PBC is associated with fatigue and post-exertional malaise, also signifying ME/CFS. We herein have investigated whether ME/CFS patients have autoreactive antibodies that could interfere with mitochondrial function. We found that only 1 of 161 examined ME/CFS patients was positive for anti-PDC, while all PBC patients (15/15) presented significant IgM, IgG, and IgA anti-PDC reactivity, as previously shown. None of fibromyalgia patients (0/14), multiple sclerosis patients (0/29), and healthy blood donors (0/44) controls showed reactivities. Anti-mitochondrial autoantibodies (inner and outer membrane) were negative in ME/CFS cohort. Anti-cardiolipin antibody levels in patients did not differ significantly from healthy blood donors. In conclusion, the impaired mitochondrial/metabolic dysfunction, observed in ME/CFS, cannot be explained by presence of circulating autoantibodies against the tested mitochondrial epitopes.

## Introduction

Myalgic encephalomyelitis (ME), also called chronic fatigue syndrome (CFS), or systemic exertional intolerance disease (SEID), is a common debilitating disease of unknown etiology characterized by post-exertional malaise (PEM), cognitive disturbance, unrefreshing sleep, autonomous nerve dysfunction and other characteristic comorbidities (1, 2). The disease may affect 0.1–0.4% of the population according to the Canadian consensus criteria (3). The biology of ME/CFS is complex and diverse explanatory models for ME/CFS have been proposed include autoimmunity, chronic infection, energy metabolic defect, imbalance in autonomous nervous system and/or hormones, and psychosomatic dysfunction.

Accumulating evidence are pointing toward an autoimmune phenotype for ME/CFS. The presence of self-reacting antibodies in the circulation of patients include nuclear and membrane structures, neurotransmitters and their receptors, neo-autoantigens formed by oxidative or nitrosative damage, and autoantibodies targeted to mitochondrial components (Table 1). However, both the frequency and the titers of autoantibodies and their correlation to disease severity or symptoms has had limited reproducibility between different studies and patient cohorts. Still, a subset of ME/CFS patients presented amelioration of symptoms following antibody removal treatment (18). Specific changes in the proteome of CSF of ME/CFS patients involved the accumulation of complement components, which signify antibody activity (19). In a recent publication from our group, the serological profile of the same ME/CFS patient cohorts demonstrated evidence of minor alterations of antibody reactivities against the ubiquitous herpesviruses when compared to healthy controls (20). These alterations may indicate shortcomings in humoral responses in ME/CFS which are hallmarks of autoimmune diseases.

**Table 1:** Autoantibodies recorded in Myalgic Encephalomyelitis/Chronic Fatigue Syndrome patients.

**Target structure**	**Autoantigen**
Neurotransmitter	5-hydroxytryptamine (4, 5)
Neurotransmitter receptors	Muscarinic M1 acetylcholine receptor (6) Muscarinic M3/4 acetylcholine receptor ß2 adrenergic receptor (7) μ-opioid receptor, 5-hydroxytryptamine receptor, dopamine receptor D2 (6)
Cell nucleus	Nuclear envelope (8, 9) Single-stranded DNA (10) Double-stranded DNA (4)
Cytoplasmic membrane	Intermediate filaments (8) Phosphatidylserine (4) Phospholipids, gangliosides (5)
Mitochondria	Heat shock protein 60 (11) Cardiolipin (4, 12, 13)
Neo-antigens	Oleic acid (14) Palmitic acid, myristic acid, S-farnesyl-L-cysteine, malondialdehyde, azelaic acid (15) NO-tyrosine (14, 15) NO-phenylalanine (14, 15) NO-arginine, NO-tryptophan, NO-cysteinyl (14) NO-Bovine serum albumin (16) NO-histidine, NO-creatine, NO-asparagine (15)
Other targets	dUTPase (17) Endothelial cells, neuronal cells (4)

*NO-, nitrosylated; dUTPase, deoxyuridine 5′-triphosphate nucleotide hydrolase*.

Recent reports point toward a central metabolic defect in ME/CFS, which affects aerobic energy production *via* the tricarboxylic acid (TCA) cycle in mitochondria, leading to a diminished production of adenosine triphosphate (ATP) and excessive lactate generation upon exertion, possibly explaining PEM (21, 22). The transition between anaerobic and aerobic energy production is catalyzed by the pyruvate dehydrogenase complex (PDC). Autoantibodies specific for PDC is a hallmark of primary biliary cholangitis (PBC), a potential disease model of autoantibody-mediated energy blockade (23, 24). In analogy with PBC, in which energy production is inhibited by antibodies (25), circulating energy inhibitors have also been detected in ME/CFS (21), however, their molecular nature is unknown. It would be reasonable if these circulating inhibitors turned out to be immunoglobulins, presumably directed against mitochondrial antigens. We have therefore investigated the presence of anti-mitochondrial antibodies and anti-PDC reactive autoantibodies, in ME/CFS patients.

## Methods

### Participants

All ME/CFS patients included in this study were diagnosed according to the Canadian consensus criteria (3). ME/CFS patients reported impairment was assessed by the Fibro-fatigue scale (26). Blood samples were acquired from three ME/CFS cohorts. Cohort 1 (*n* = 74): 46 ME/CFS patients, 17 ME/CFS + fibromyalgia (FM) patients, and 11 FM patients. This cohort also included 29 multiple sclerosis (MS) patients. Cohort 2 (*n* = 61): 61 ME/CFS patients; Cohort 3 (*n* = 40): 18 ME/CFS patients, 19 ME/CFS/FM patients, 3 FM patients, and 15 age-matched healthy donors in cohort 3 (HD3). Samples from cohorts 1–3 originated from the Gottfries Clinic, Mölndal, Sweden. The characteristics of the patients are summarized in Table 2. Plasma samples from 15 PBC patients were collected at the blood bank of The Medical School in The University of Newcastle upon Tyne, UK. Additional controls included serum samples from 46 anonymous healthy blood donors from Uppsala Academic Hospital University, Sweden.

**Table 2A:** Characteristics of patient study cohorts 1 and 2.

		**ME/CFS**		**ME/CFS+FM**	**FM**
Demographics	Cohort # Sex (female/male) Age, mean ± SD (years)	#1 (*n* = 46) (34/12) 45.8 ± 9.2	#2 (*n* = 61) (51/10) 46.9 ± 11.0	#1 (*n* = 17) (14/3) 44.5 ± 9.7	#1 (*n* = 11) (8/3) 46.8 ± 10.7
Severity	Disease duration mean ± SD (years) Fibro-fatigue sum score mean ± SD (range: 0–72)	11.7 ± 7.7 40.0 ± 9.1	8.6 ± 10.0 35.5 ± 7.8	11.7 ± 7.7 40.0 ± 9.1	14.4 ± 10.1 40.0 ± 13.5

**Table 2B:** Characteristics of patient study cohort 3.

		**ME/CFS, ME/CFS+FM, FM**
Demographics	Cohort # Sex (female/male) Age, mean ± SD (years)	#3 (*n* = 37) (26/11) 42 ± 12
Severity	Disease duration mean ± SD (years) Fibro-fatigue sum score mean ± SD (range: 0–72) Work disability %	9 ± 5 N/A 70% (26/37)
Trigger event	Infectious %	81% (30/37)

*SD, Standard Deviation, N/A, Not Available. ME/CFS, myalgic encephalomyelitis/chronic fatigue syndrome; FM, fibromyalgia*.

### Ethics Statement

Informed consent for blood collection was obtained from all patients according to the Declaration of Helsinki, and ethical approval was granted by the ethical review committee at Medical Faculty, University of Gothenburg Dnr 029-13, Dnr 867-13, T 091-16, and Dnr 852-12, T 092-16 (CGG). Blood donor samples were collected anonymously according to ethical consent from the Uppsala Institutional Review Board (IRB) UPS_01_367, and Linköping Regional Office of Swedish Ethical Review Authority. The 15 PBC patient samples from Newcastle upon Tyne, were under REC number 12/NE/0095.

### Detection of Anti-PDC Antibodies

Antibodies against human PDC (hPDC) were measured by in-house ELISA, as previously described (27). Briefly, wells of *Immulon 4HBX* microtiter plates (Dynex Technologies Inc., El Paso, TX) were coated with 2.5 μg/ml hPDC in 50 mM NaHCO_3_/Na_2_CO_3_ (pH 9.6). The plates were blocked with 5% (w/v) bovine serum albumin (BSA) in phosphate-buffered saline (PBS), and incubated with plasma diluted 1:500 in PBS/0.5% (w/v) BSA for 3 h. Specific antibody binding was detected with goat anti-human IgG, IgM, or IgA heavy chain specific peroxidase conjugates (Sigma, Poole, UK) and o-phenylenediamine dihydrochloride (OPD). Absorbance was recorded at 492 nm, and any values over 0.370 nm (mean OD value +3 SD) were regarded as positive for IgG anti-PDC, 0.211 nm for IgM and 0.0152 nm for IgA.

### Validation of Anti-PDC Antibodies

Extracted human PDC was resolved with 10% SDS PAGE and immunoblotting was performed as previously described (24, 27). The membrane was blocked with 5% (w/v) skimmed milk powder and then probed with patient plasma from three ME/CFS and one ME/CFS/FM, all diluted 1:500 in 0.5% w/v of BSA in PBS–T. Bound antibodies were detected using goat anti-human IgG peroxidase-conjugated antibodies (Sigma, Poole, UK) and enhanced chemiluminescence (ECL; Amersham, Aylesbury, UK) according to the manufacturer's protocol.

### Detection of Anti-mitochondrial Autoantibodies (AMA)

Plasma samples from 29 randomly selected ME/CFS patients were screened using Multi-spot slides, with fixed tissue sections (NOVA Lite® Rat Stomach, Liver, Kidney, San Diego, CA). All procedures were performed according to the manufacturer's instructions as part of a validated method used for diagnostic purposes in Linköping University Hospital, Sweden.

## Detection of Anti-cardiolipin Autoantibodies (ACA)

IgG and IgM anti-cardiolipin antibodies were analyzed in the Clinical Immunology Laboratory, Linköping University Hospital, Sweden using the ELiAtex™ cardiolipin IgG and IgM fluoro-enzyme-immunoassay with the Phadia-250 instrument (Thermo-Fisher Scientific Phadia AB, Uppsala, Sweden). For IgA anti-cardiolipin antibodies, samples were examined with the Inova QUANTA Lite ACA IgA III ELISA (Inova, Altenhostrasse, Germany). Both assays were done according to manufacturer's instructions.

### Statistical Analysis

For the comparison between multiple patient groups, we used one-way analysis of variance (ANOVA) using GraphPad Prism version 6.0 software (GraphPad Software, San Diego, CA, USA). Only statistically significant differences *p* < 0.05 were reported.

## Results

### Autoantibodies Against Mitochondrial Pyruvate Dehydrogenase Complex (PDC) Not Detected in ME/CFS

**Figure 1:**
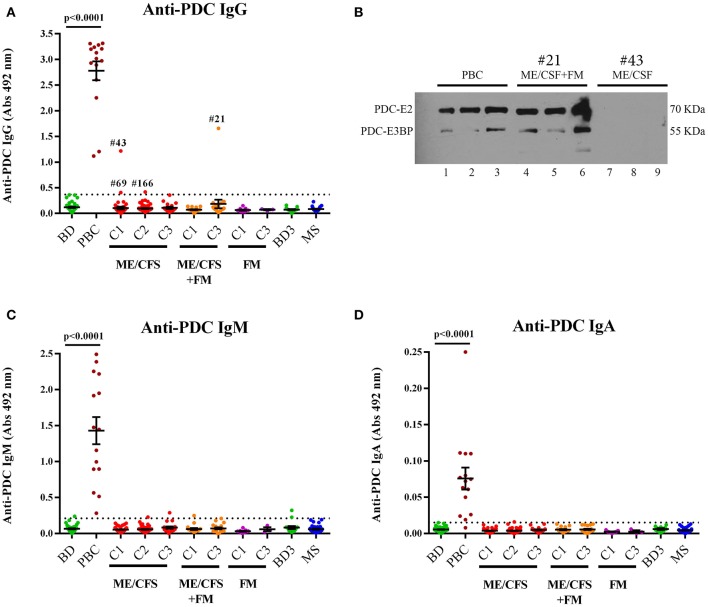
**(A)** Anti-pyruvate dehydrogenase complex (anti-PDC) IgG antibody levels in the plasma of healthy blood donors (BD, *n* = 44), primary biliary cholangitis patients (PBC, *n* = 15), myalgic encephalomyelitis/chronic fatigue syndrome (ME/CFS) patients (cohort 1: C1, *n* = 46, cohort 2: C2, *n* = 61 and cohort 3: C3, *n* = 18), myalgic encephalomyelitis/chronic fatigue syndrome with fibromyalgia (ME/CFS+FM) (cohort 1: C1, *n* = 17 and cohort 3: C3, *n* = 19), fibromyalgia (FM) patients (cohort 1: C1, *n* = 11 and cohort 3: C3, *n* = 3), age-matched healthy blood donors (BD3, *n* = 15) from cohort 3, and multiple sclerosis (MS) patients (cohort1: C1, *n* = 29). **(B)** Immunoblot analysis against human extracted pyruvate dehydrogenase of pooled primary biliary cholangitis (PBC) serum (lanes 1–3), plasma sample from patient #21 with myalgic encephalomyelitis/chronic fatigue syndrome with fibromyalgia (ME/CFS+FM patient (lanes: 4–6), and plasma sample from patient #43 with myalgic encephalomyelitis/chronic fatigue syndrome patient (lanes: 7–9). Samples were found positive for anti-PDC antibodies using the anti-PDC ELISA. Pooled PBC sample and patient #21 (ME/CFS+FM) were found positive for the major autoreactive proteins within the PDC complex, dihydrolipoamide acetyltransferase (E2) and the E3-binding protein (E3BP), whereas patient #43 (ME/CFS) was found negative. **(C,D)** Anti-PDC antibody levels of IgM and IgA subclass in the same samples as in panel **(A)**. Dotted line represents cut-off levels for each Immunoglobulin subclass. Data are represented as individual points for each subject, overlaid by a horizontal mean group value ± SEM. Multiple group comparisons were analyzed by one-way ANOVA. Statistically significant differences are indicated (*p* < 0.0001).

**Figure 2:**
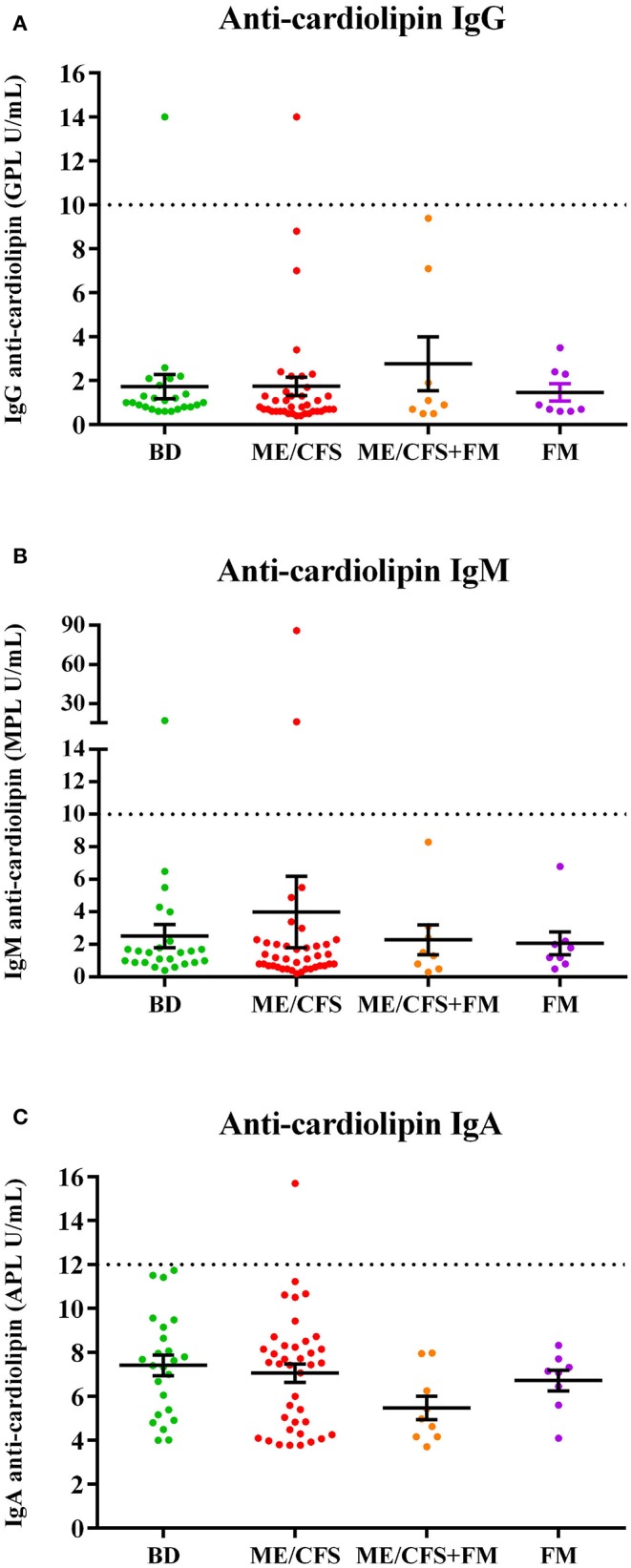
Anti-cardiolipin (ACA) antibody levels of IgG **(A)**, IgM **(B)**, and IgA **(C)** subclasses in the plasma of healthy blood donors and patients in cohort 1. For IgG and IgM the analysis included samples from healthy blood donors (BD, *n* = 24), myalgic encephalomyelitis/chronic fatigue syndrome (ME/CFS patients, *n* = 39), myalgic encephalomyelitis/chronic fatigue syndrome with fibromyalgia (ME/CFS+FM, *n* = 8) and fibromyalgia (FM, *n* = 8) patients. For IgA ACA analysis samples included BD (*n* = 24), ME/CFS patients (*n* = 39), ME/CSF+FM patients (*n* = 9), and FM (*n* = 8) patients. GPL, MPL, and APL correspond to IgG, IgM and IgA phospholipid units, respectively. Dotted lines represent cut-off levels for each subclass. Data are represented as individual points for each subject, overlaid by a horizontal mean group value ± SEM. For IgG and IgM ACA antibody levels, values < 10 U/mL were considered negative, values between 10 and 40 were considered as weak positive and values higher that 40 were considered positive. For IgA ACA, values < 12 U/mL were considered negative. No statistically significant differences were noted.

In this study, we analyzed whether dysfunctional energy generation from mitochondria in ME/CFS could be explained by the presence of reactive autoantibodies directed against the PDC enzyme, in analogy to what has been observed in PBC. ME/CFS, PBC, FM, MS and healthy blood donor controls were analyzed (Figure 1). PBC patient plasma samples were all positive for IgG, IgM, and IgA anti-PDC antibodies and hence presented with statistically significant differences (*p* < 0.0001). In repeated analyses, these samples remained positive down to dilutions of 1:10^5−^1:10^6^. In contrast to our hypothesis, ME/CFS samples were negative for autoantibodies against PDC, with the exception of three ME/CFS patients: #43 (A = 1.217 at 1:500); ME/CFS patient #69 (A = 0.406 at 1:500); ME/CFS patient #166 (A = 0.418 at 1:500); and #21, a ME/CFS patient with FM comorbidity (A =1.658 at 1:500) that was found weakly/intermediately positive. These plasma samples were tested additionally by immunoblot analysis for validation. Only sample from patient #21 (ME/CFS+FM) was positive, whereas the other samples were negative (Figure 2). Autoreactivity was identified against the major autoreactive epitopes within the PDC complex, dihydrolipoamide acetyltransferase (E2) and the E3-binding protein (E3BP) as previously described (24).

### AMA and ACA Not Detected in ME/CFS Patients

The ME/CFS patient group did not exhibit AMAs, against inner and outer mitochondrial membrane, nor antibodies against other mitochondrial structures, nor other structures (nuclear, smooth muscle, gastric parietal cell). Anti-cardiolipin antibody (IgG, IgM, IgA) levels were not significantly different in plasma of ME/CFS or FM patients compared with blood donors (Figure 2).

## Discussion

We conclude from the results presented in this study, that in contrast to our initial expectation, ME/CFS patients do not have autoantibodies to epitopes of mitochondrial components. This does not rule out that other important indirect or secondary mechanisms exist in blocking the complex mitochondrial pyruvate metabolism in ME/CFS.

An example of autoimmunity with autoreactive antibodies against a key metabolic factor is PBC. It was previously shown that anti-PDC antibodies in these patients are mainly directed against the inner lipoyl domain of the PDC-E2 component, which has an alpha-lipoic acid covalently bound to a specific lysine residue, which is an absolute requirement for its enzymatic activity. Lipoylation is a posttranslational modification, which also occurs in bacteria like *E. coli* or *Novosphingobium* sp. (28). Autoreactive responses in PBC have been suggested to arise after infection in the gut with these bacteria (29, 30) or beta-retroviruses (31), or exposure to environmental xenobiotics that mimic the native lipoic acid moiety (32). PBC patients have a PEM reminiscent of PEM in ME/CFS and FM patients (33). FM is a common comorbidity in both ME/CFS, occurring in ~30–70% of the patients, and autoimmunity (34–36). Metabolic disturbances in ME/CFS with reduced energy supply and increased lactate may account for most of the malaise in PEM as well as cognitive and physical disturbances (37). Notably, accumulation of lactate in blood and muscles after exercise, along with increased concentrations of lactate in cerebrospinal fluid (CSF) have been found in ME/CSF (38, 39). However, our present comparative analysis for AMA, anti-PDC, and anti-cardiolipin autoantibodies in the 3 cohorts did not yield a positive or disease-specific result.

A link between mitochondrial dysfunction and innate immune dysregulation is suggested which demonstrate that the energy producing organelles (mitochondria and peroxisomes) are coupled via mitochondrial antiviral signaling proteins (MAVS) to the inflammasome (40). Hypothetically, danger-associated molecular patterns (DAMPs), such as mitochondrial DNA (mtDNA), heat shock proteins (Hsps), and cardiolipins could be released from the mitochondria (41–43) in ME/CFS and act as autoantigens. We previously found that a subset of ME/CFS patients had higher levels of IgM antibodies against epitopes of both mitochondrial and bacterial Hsp-60 (despite the absence of an infectious pathogen) which potentially may lead to dysfunctional mitochondria (11). Our hypothesis on the presence of autoantibodies against cardiolipin in ME/CFS patients was based on previous publications (ref in Table 1), could not be confirmed here, possible explained by different inclusion and exclusion criteria of patients in previous studies that did not used Canadian criteria, but included leukemia/lymphoma and type 1 diabetes patients. Further studies are required to analyse unique epitope alterations such as oxidation epitopes of cardiolipin (42), length and 3D-structure, which each may signal differently in this complex immune-metabolic scenario.

The hypothesis of a direct blocking of PDC due to autoreactive antibodies is excluded based on our present findings, however, blocking could be secondary and a sign of compensatory efforts to balance some other abnormalities in ME/CFS, as suggested by Fluge et al. (21). Their data indicate a functional impairment of PDC leading to increased consumption of specifically ketogenic and anaplerotic amino acids that fuel alternative pathways for ATP production independently of PDC. The results were supported by increased mRNA expression of pyruvate dehydrogenase kinases that are inhibitory to PDC (21). Recent studies also show that many fatty acids may generate acetyl-CoA that fuel the TCA-cycle independently of PDC (44). Similar observations on altered energy metabolism may occur in different types of cellular stress such as starvation (45). Blocking of PDC could also be generated by other mechanisms including reduction-oxidation (redox) reactions known to modulate lipoic acid moiety in the PDC (46). Therefore, it would be of interest to investigate in future studies the PDC redox status in ME/CFS patients. Finally, multiple enzymes such as mitochondria pyruvate carrier, pyruvate carboxylases, PD-kinases, PD-phosphatases, in addition to PDC, modulate overall pyruvate carbon flux (47).

## Data Availability Statement

The datasets generated for this study are available on request to the corresponding author.

## Ethics Statement

The studies involving human participants were reviewed and approved by the ethical review committee at Medical Faculty, University of Gothenburg; Uppsala Institutional Review Board; Linköping Regional Office of Swedish Ethical Review Authority, Newcastle upon Tyne, under REC number 12/NE/0095; Regional ethic committee of Stockholm. The patients/participants provided their written informed consent to participate in this study.

## Author Contributions

IN, JP, and EA designed and performed experiments, analysis, and authored the manuscript. MR and C-GG provided critical materials. CD performed experiments and analysis. AR conceived the design and execution of all experimental procedures and authored the manuscript. All authors critically reviewed the final manuscript.

### Conflict of Interest

C-GG was employed by the Gottfries Clinic AB company. The remaining authors declare that the research was conducted in the absence of any commercial or financial relationships that could be construed as a potential conflict of interest.
